# The Medico-Psychological Society and Mental Nurses

**Published:** 1897-04-17

**Authors:** 


### The Institutional Workshop.

### THE MEDICO-PSYCHOLOGICAL SOCIETY AND MENTAL NURSES.

In illustration of our contention that grave dangers are inherent in the system by which nursing certificates are granted, and also to emphasise our position that the practical work done during the training should have a greater value put upon it, we append quotations which we have extracted from the pages of the Journal of the Medico-Psychological Association itself. From these it will be seen that we are not singular in the opinions we expressed, nor without some warrant for them. The status of the Medico-Psychological certificate will depend, not upon the character of those exceptionally well-trained nurses who hold it, but upon the minimum amount of knowledge which has been necessary to qualify for it. How much that may be we leave our readers to judge from the opinions of prominent members of the association.

Dr. Macdonald (Superintendent of Dorset County Asylum), in a thoughtful and sympathetic paper on the nursing staff of asylums, says, "Whereas the so-called Association medal is given for answering a few questions, which is no guarantee of a thorough nurse or attendant, I am in favour of teaching and training the staff. I have already said that such is an integral part of our system, but I wish to sound a note of warning against the prevalent and rather absurd practice of recognising these parchments as proofs of efficiency."

At a meeting of the Irish division of the Association held in Dublin in May, 1896, the training of asylum attendants was considered.

Dr. Finegan (Superintendent of Mullingar District Asylum) introduced the subject, dealing especially with the way in which papers were examined. He thought that too many of the candidates who presented themselves were allowed to pass. In some of the results announced recently he noticed that all the candidates succeeded. He feared that there was an unwillingness to "stick" candidates. As far as the Mullingar Asylum was concerned, all that went up were able to read and write, and passed. (Laughter.) He suggested that they should consider whether it would not be advisable that an Educational Committee should be appointed who would either mark the papers or select examiners for the purpose.

Dr. Woods (Superintendent of Cork District Asylum) said that he had raised this question in London at the annual meeting, and he quite agreed with the views of Dr. Finegan in favour of common examiners for all the papers. The duty of examining all the papers should be referred to one person or an examining board, and no candidate should be allowed to pass who did not come up to the common standard. Dr. Finegan's statement about all being allowed to pass was hardly borne out by the facts, which showed the proportion of failures to be about 28 per cent., at all events in Cork and Limerick. In some asylums he feared there was a system of cram, and he thought they ought to extend the lectures over a long period. Those who did not intend going for the next examination should be compelled to attend, and promotion should only be given to those who passed the examination.

Dr. O'Neill (Superintendent, Limerick District Asylum) agreed with what Dr. Finegan had said about the necessity for greater care in the examinations. It certainly seemed strange to say that out of twenty or twenty-one names in one asylum not one was stopped. Marks ought be given to attendants and nurses for general conduct, condition of their wards, appearance of patients, and general character. Of course, if that could not be done, some provision should be made to recognise these evidences of care in duty.

The matter was brought before the annual meeting in London, July, 1896.

Examination Papers for the Nursing Certificate.

A discussion then ensued on the resolution passed at the Irish Divisional Meeting on May 7th. It is as follows: "In the opinion of this meeting it is desirable that the next annual meeting should consider the propriety of arranging that in future the papers issued to the candidates for the nursing certificate be valued and marked by the examiners in each division of the kingdom."

Dr. Oscar Woods said the resolution was not passed by the Irish division without a good deal of discussion, part of which had appeared in the Journal. It was admitted by all, he thought, and even by many English members, that considerable improvement might be made in the mode of examining attendants for the nursing certificate. The resolution implied that the test was not a uniform one. He moved that the subject of the amendment of the examination for the nursing certificates be referred to the Educational Committee with a strong expression of feeling both from the Council and this meeting that the matter is an urgent one, and that everything possible be done to secure that the examination should be a uniform test.

Dr. Spence (Superintendent Stafford County Asylum) did not wish that discussion should be burked. If there were any improvements which should be introduced in the mode of conducting the examinations he would be only too willing to join with those who wished them. It was not a personal matter as far as he was concerned; but he thought it a reflection on the medical superintendents of the United Kingdom who had examined candidates to be practically told that they let people through whom they had no business to pass.

Dr. Yellowlees (Superintendent, Glasgow Royal Asylum, Gartnavel) agreed with Dr. Newington. He was quite sure they never would have the examinations satisfactory with regard to results until the written papers were examined by those who set them. He thought it no reflection on medical superintendents and assessors to take this step, as, while human nature remained what it was, there would be men who would pluck their neighbours' attendants. As a matter of fact the examination for the certificate in its results was the most unequal he had known. He thought it might be added that the matter was urgent, and the Educational Committee might consider it at that meeting. Much more might be said profitably on the matter than could be openly said, and from what he knew he believed there was great need for something to be done.

It was therefore agreed to pass the motion as follows: "That the subject of the management of the examination for the Nursing Certificate be referred to the Educational Committee with an expression of feeling from the association that the question is an urgent one."

We refrain from comments, though some of the expressions used are, to say the least, very suggestive.

